# Activation of AMP-Activated Protein Kinase Is Required for Berberine-Induced Reduction of Atherosclerosis in Mice: The Role of Uncoupling Protein 2

**DOI:** 10.1371/journal.pone.0025436

**Published:** 2011-09-27

**Authors:** Qilong Wang, Miao Zhang, Bin Liang, Najeeb Shirwany, Yi Zhu, Ming-Hui Zou

**Affiliations:** 1 Section of Molecular Medicine, Department of Medicine, University of Oklahoma Health Sciences Center, Oklahoma City, Oklahoma, United States of America; 2 Physiology and Pathophysiology, Peking University Health Science Center, Beijing, China; 3 Department of Biochemistry and Molecular Biology, University of Oklahoma Health Sciences Center, Oklahoma City, Oklahoma, United States of America; University of Hong Kong, China

## Abstract

**Aims:**

Berberine, a botanical alkaloid purified from Coptidis rhizoma, is reported to activate the AMP-activated protein kinase (AMPK). Whether AMPK is required for the protective effects of berberine in cardiovascular diseases remains unknown. This study was designed to determine whether AMPK is required for berberine-induced reduction of oxidative stress and atherosclerosis *in vivo*.

**Methods:**

ApoE (ApoE^-/-^) mice and ApoE^-/-^/AMPK alpha 2^-/-^ mice that were fed Western diets were treated with berberine for 8 weeks. Atherosclerotic aortic lesions, expression of uncoupling protein 2 (UCP2), and markers of oxidative stress were evaluated in isolated aortas.

**Results:**

In ApoE^-/-^ mice, chronic administration of berberine significantly reduced aortic lesions, markedly reduced oxidative stress and expression of adhesion molecules in aorta, and significantly increased UCP2 levels. In contrast, in ApoE^-/-^/AMPK alpha 2^-/-^ mice, berberine had little effect on those endpoints. In cultured human umbilical vein endothelial cells (HUVECs), berberine significantly increased UCP2 mRNA and protein expression in an AMPK-dependent manner. Transfection of HUVECs with nuclear respiratory factor 1 (NRF1)-specific siRNA attenuated berberine-induced expression of UCP2, whereas transfection with control siRNA did not. Finally, berberine promoted mitochondrial biogenesis that contributed to up-regulation of UCP2 expression.

**Conclusion:**

We conclude that berberine reduces oxidative stress and vascular inflammation, and suppresses atherogenesis via a mechanism that includes stimulation of AMPK-dependent UCP2 expression.

## Introduction

Berberine is an isoquinolone alkaloid found in plants such as *Phellodendron chinense* and *Coptis chinensis.* In traditional Chinese medicine, berberine from *Coptidis rhizoma* is used as a constituent of the herbal medicine Huanglian. In this form it is reported to exert anti-fungal, anti-bacterial/viral, and anti-oncogenic effects, as well as a beneficial effect on diabetes [1], [2], [3], [4], [5], and anti-atherogenic properties [6], [7], [8]. Berberine has also been reported to reduce the incidence of cardiovascular diseases [9], [10], [11]. Several mechanisms are reported to be associated with the beneficial properties of berberine including improvement of endothelial function and dyslipidemia, inhibition of low-density lipoprotein oxidation, and decreased blood pressure. However, the molecular targets through which berberine exerts its beneficial effects are undefined.

AMP-activated protein kinase (AMPK) is a heterotrimer consisting of α, β, and γ subunits, which each have at least two isoforms. The α subunit contains the catalytic site, but all subunits are necessary for full activity. An increase in the ratio of AMP/ATP activates AMPK by a number of mechanisms, including direct allosteric activation and covalent modification in which an AMP-dependent AMPK kinase (AMPKK) phosphorylates the α subunit on Thr172. AMPK is generally quiescent under normal conditions but is activated in response to signals and stresses that increase the AMP/ATP ratio, such as hypoglycemia, strenuous exercise, anoxia, and ischemia. Once activated, AMPK switches on catabolic pathways that generate ATP, while switching off ATP-consuming processes (e.g., biosynthesis, cell growth, and proliferation), and in doing so acts as an “energy gauge”. This homeostasis function is regarded as a fundamental feature of multiple AMPK-mediated biological processes [12].

Although the effects of AMPK on key metabolically relevant organs and tissues such as liver, skeletal muscle, adipose tissue, and hypothalamus have been relatively well documented [13], the notion that AMPK activation could be used to promote vascular health has only recently emerged. For example, in vascular tissues AMPK activation appears to be a shared molecular target [14] for the beneficial effects of several interventions including physical exercise [15], statins, thiazolidinediones (TZDs) [16], leptin [17], [18], adiponectin [19], [20], [21], and rosiglitazone in diabetes [22], [23], [24]. AMPK is also important for maintaining endothelial cell nitric oxide (NO) activity and endothelial function via the AMPK-endothelial nitric oxide synthase (eNOS)-NO axis [25]. Moreover, AMPK activation might indirectly improve vascular endothelial function by improving metabolic profiles and insulin sensitivity [26], [27], [28].

Recent studies, including studies from our group, have shown that berberine causes a dose- and time-dependent activation of AMPK in cultured endothelial cells [29], [30], [31]. In isolated aortas, berberine-dependent AMPK activation improves endothelium-dependent vasorelaxation, likely via AMPK-dependent phosphorylation of endothelial nitric oxide synthase [32]. Although beneficial effects of berberine in cardiovascular diseases have been reported [10], [33], to date no study has examined the effects of chronic administration of berberine on atherosclerosis *in vivo*. Furthermore, it is important to determine whether AMPK activation is required for anti-atherosclerotic effects of berberine *in vivo* in order to comprehensively elucidate its properties. The present study investigated the effects of berberine on atherosclerosis and the relative contributions of AMPK alpha 2 in berberine-induced suppression of atherosclerotic lesions.

## Materials and Methods

### Materials

Antibodies against phospho-AMPK, intercellular adhesion molecule (ICAM)-1, vascular cell adhesion molecule (VCAM)-1, 4-hydroxy-trans-2-nonenal (4-HNE), 3-nitrotryosine (3-NT) and β-actin were obtained from Cell Signaling Biotechnology (Danvers, MA). Antibodies against uncoupling protein (UCP) 2 were obtained from Fitzgerald Industries International, Inc. (Acton, MA). Nuclear respiratory factor (NRF)1 and mitochondrial transcription factor A (mtTFA) antibodies, and the specific siRNA for AMPK were purchased from Santa Cruz Biotechnology Inc. (Santa Cruz, CA). AICAR was from Toronto Research Chemicals Inc. (North York, ON, Canada), and all other chemicals, unless indicated, were purchased from Sigma-Aldrich (St. Louis, MO).

### Animals

Mice were housed in temperature-controlled cages under a 12-h light-dark cycle with free access to water and either regular rodent diet *ad libitum* or a modified pro-atherogenic diet as indicated. AMPK alpha 2^-/-^ mice [5], [34] that had been backcrossed onto a wild-type C57BL/6 background were crossed with C57BL/6 ApoE^-/-^ mice (The Jackson Laboratory, Bar Harbor, ME) to generate ApoE^-/-^/AMPK alpha 2^-/-^ mice. Accelerated atherosclerosis was induced by feeding the mice a Western diet containing 0.21% cholesterol and 21% fat (D12079B, Research Diets Inc,). This diet was administered beginning at 5 weeks of age and continued for 8 consecutive weeks. At 5 weeks of age, berberine (Sigma-Aldrich) was also added to the drinking water (1 mmol/L) for 8 weeks. The animal protocol (10-142H) was reviewed and approved by the University of Oklahoma Institutional Animal Care and Use Committee (IACUC).

### Cell culture

Human umbilical vein endothelial cells (HUVECs) obtained from ATCC (Manassas, VA) were grown in Eagle basal medium (Clonetics Inc, Walkersville, MD) supplemented with 5% fetal bovine serum (FBS), penicillin (100 µg/mL), and streptomycin (100 µg/mL). Cells between passages 3 and 8 were used in all experiments. Cells were incubated at 37°C in a humidified atmosphere of 5% CO_2_ and 95% air, and grown to 70% to 80% confluence before being treated with the indicated agents.

### Determination of serum cholesterol, triglyceride, and blood glucose levels

Blood glucose levels from non-fasting mice were determined by applying tail blood to an OneTouch Ultra Blood Glucose Monitoring System (LifeScan). Serum cholesterol and triglyceride levels were measured enzymatically, using Infinity reagents (Thermo DMA) according to the manufacturer's instructions.

### Atherosclerotic lesion analysis

After being fed a Western diet for 8 weeks, mice were anesthetized and euthanized. The heart and aortic tissue were removed from the ascending aorta to the iliac bifurcation and placed in 4% paraformaldehyde for 16 h. For analysis of the lesion area in the aortic arch, the intimal surface was exposed by a longitudinal cut from the ascending arch to 5 mm distal of the left subclavian artery to allow the lumen of the aortic arch to be laid flat. The aorta was rinsed for 5 min in 75% ethanol, stained with 0.5% Sudan IV in 35% ethanol and 50% acetone for 15 min, destained in 75% ethanol for 5 min, and then rinsed with distilled water. Digital images of the aorta were captured under a stereomicroscope, and the lesion area was quantified from the aortic arch to 5 mm distal of the left subclavian artery using Alpha Ease FC software (version 4.0 Alpha Innotech). To evaluate plaque formation in other parts of the aorta, 8-µm-thick cross-sections of atherosclerotic lesions from the proximal aorta were stained with hematoxylin and eosin after fixation in 4% paraformaldehyde.

### Immunocytochemistry staining

Immunocytochemistry staining and analysis of aortic lesions were performed as described previously [35].

### Real-time PCR analysis

Total mRNA was isolated from HUVECs using the QIAGEN® RNeasy® Mini kit. UCP2 mRNA was quantified by real-time PCR as described previously [36].

### Western blot analysis

Cell lysates and tissue homogenates were subjected to Western blot analysis as described previously [37].

### Statistics

Results were analyzed with a one-way ANOVA with appropriate post-hoc analysis of results and Student's *t* test. Values were expressed as mean ± SE. A *p* value less than 0.05 was considered statistically significant.

## Results

### Chronic administration of berberine significantly attenuates aortic lesions in ApoE^-/-^ mice but not AMPK alpha 2^-/-^/ApoE^-/-^ mice

**Figure 1:**
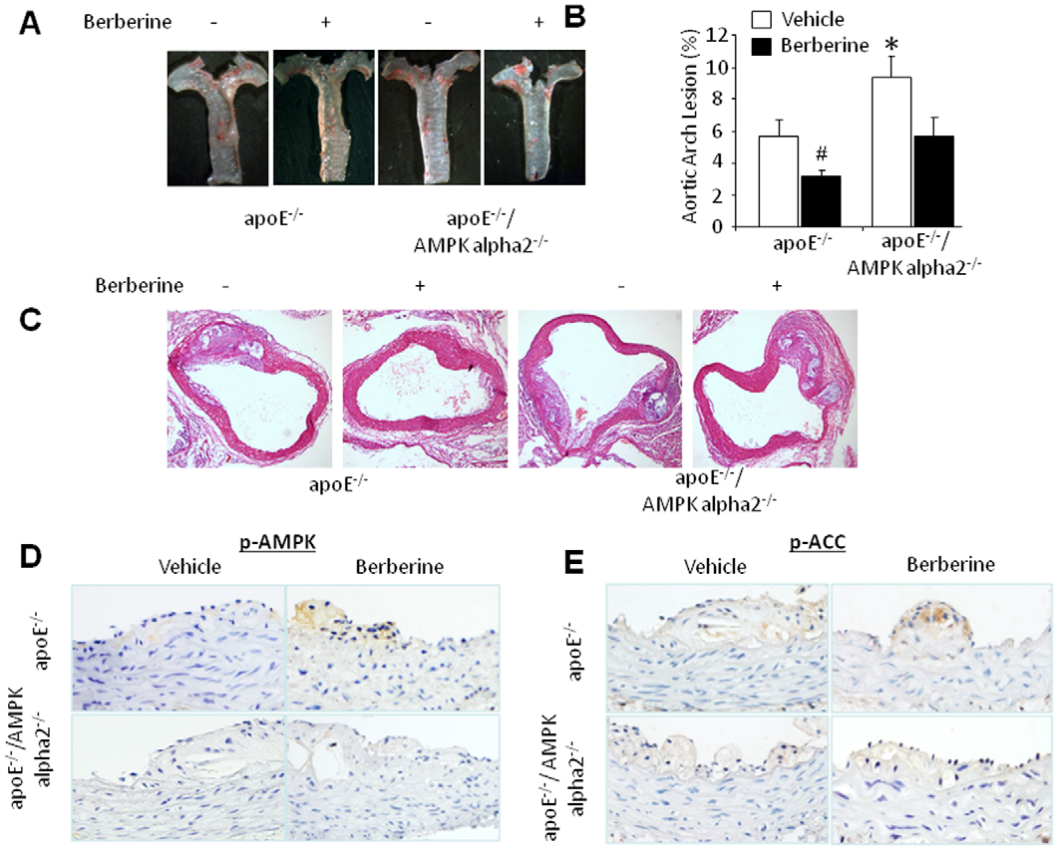
AMPK-dependent reduction of aortic lesions by berberine *in vivo.* **A.** Representative images of aortic arch lesion in ApoE^-/-^ and ApoE^-/-^/AMPK alpha 2^-/-^ mice fed a HFD. Berberine reduced the size of lesions in both ApoE^-/-^ and ApoE^-/-^/AMPK alpha 2^-/-^ mice. **B.** Aortic arch atherosclerotic lesions were measured as percentage of luminal diameter in ApoE^-/-^ and ApoE^-/-^/AMPK alpha 2^-/-^ mice with or without berberine treatment. *p<0.05 ApoE^-/-^ *vs*. ApoE^-/-^/AMPK alpha 2^-/-^, # p<0.05 berberine *vs*. vehicle. n = 7 or 8 for each group. **C**. Proximal aorta section showing atherosclerotic lesions. Shown are representative cross-sections from serial sections of proximal aorta visualized by hematoxylin and eosin staining. In each group, 4–5 mice were evaluated and displayed similar phenotypes. **D&E.** Aortic sections from ApoE^-/-^ and ApoE^-/-^/AMPK alpha 2^-/-^ mice fed a HFD were immunohistochemically probed with antibodies specific for p-AMPK and p-ACC. n = 7 or 8 for each group.

ApoE^-/-^ mice are known to develop a robust aortic atherosclerotic phenotype when fed a Western diet [38]. Therefore, we first evaluated the effects of berberine on the severity of aortic atherosclerosis in ApoE^-/-^ and ApoE^-/-^/AMPK alpha 2^-/-^ mice maintained on this diet. ApoE^-/-^/APMK alpha 2^-/-^ mice developed more aortic lesions than ApoE^-/-^ mice (Fig. 1A-C). Interestingly, berberine treatment significantly reduced the severity of atherosclerotic lesions in the aortic arch of ApoE^-/-^ mice. The aortic lesion-mitigating effect of berberine was less strong in ApoE^-/-^/APMK alpha 2^-/-^ mice than in ApoE^-/-^ mice (Fig. 1A–C), suggesting that APMK alpha 2 is critical for the anti-atherosclerotic effects of berberine in ApoE^-/-^ mice.

### Berberine increases the phosphorylation of AMPK at Thr172 and acetyl-CoA carboxylase (ACC) at Ser79 in ApoE^-/-^aortas

The observation that berberine had a smaller effect on aortic lesions in ApoE^-/-^/AMPK alpha 2^-/-^ mice prompted us to determine whether berberine activates AMPK in the affected areas of the aorta. Because Thr172 phosphorylation of AMPK is closely associated with AMPK activation and can be used as an index for AMPK activity [25], we first monitored the levels of AMPK-Thr172 phosphorylation in aortic tissue from vehicle- or berberine-treated ApoE-/- mice using immunocytochemical (IHC) staining. Berberine markedly increased the level of p-AMPK staining in aortic tissues from ApoE^-/-^ mice, but did not increase staining in aortic tissues from ApoE^-/-^/AMPK alpha 2^-/-^ mice (Fig. 1D).

Acetyl CoA carboxylase (ACC) is a well-characterized substrate of AMPK in endothelial cells, and phosphorylation of ACC at Ser79 reflects AMPK activity in tissues [39]. We monitored p-ACC levels in aortic tissues from both ApoE^-/-^ and ApoE^-/-^/AMPK alpha 2^-/-^ mice. In accord with the p-AMPK findings, berberine treatment markedly increased the levels of p-ACC in ApoE^-/-^ mice, but not in ApoE^-/-^/AMPK alpha 2^-/-^ mice (Fig. 1E). Overall, these data suggest that berberine activates AMPK in atherosclerotic aortic tissues.

### Effects of berberine on serum cholesterol, triglycerides, and glucose

We next determined if chronic berberine administration alters the levels of lipids and glucose, which exert effecs on atherosclerotic lesions. As shown in Table 1, typical metabolic parameters (blood glucose, serum cholesterol, and triglyceride) did not differ between ApoE^-/-^/AMPK alpha 2^-/-^ mice and ApoE^-/-^ mice. In addition, berberine treatment did not affect the levels of blood glucose, cholesterol, and triglyceride in ApoE^-/-^ mice or ApoE^-/-^/AMPK alpha 2^-/-^ mice (Table 1).

**Table 1:** Serum cholesterol, triglyceride, blood glucose and body weight in ApoE^-/-^ and ApoE^-/-^/AMPK alpha 2^-/-^ mice.

	ApoE^-/-^		ApoE^-/-^/AMPK alpha 2^-/-^	
	Vehicle	Berberine	Vehicle	Berberine
Body weight (g)	25.5±1.9	26.8±1.3	28.9±2.7	27.5±1.5
Cholesterol (mmol/l)	29.5±3.6	23.4±0.8	29.5±3.0	26.6±1.1
Triglyceride (mmol/l)	1.53±0.34	1.15±0.12	1.19±0.29	0.97±0.14
Glucose (mg/dl)	224±25.6	231±16.2	178.7±16.9	170.3±16.2

Values are mean ± SE. n = 7-8 per group. Blood glucose levels were determined by applying tail blood from non-fasting mice to a One touch Ultra Blood Glucose Monitoring System (LifeScan). Serum cholesterol and triglyceride levels were measured enzymatically using Infinity reagents (Thermo DMA) according to the manufacturer's instructions.

### Berberine attenuates expression of adhesion molecules VCAM-1 and ICAM-1 in aortic atherosclerosis lesions *in vivo*

**Figure 2:**
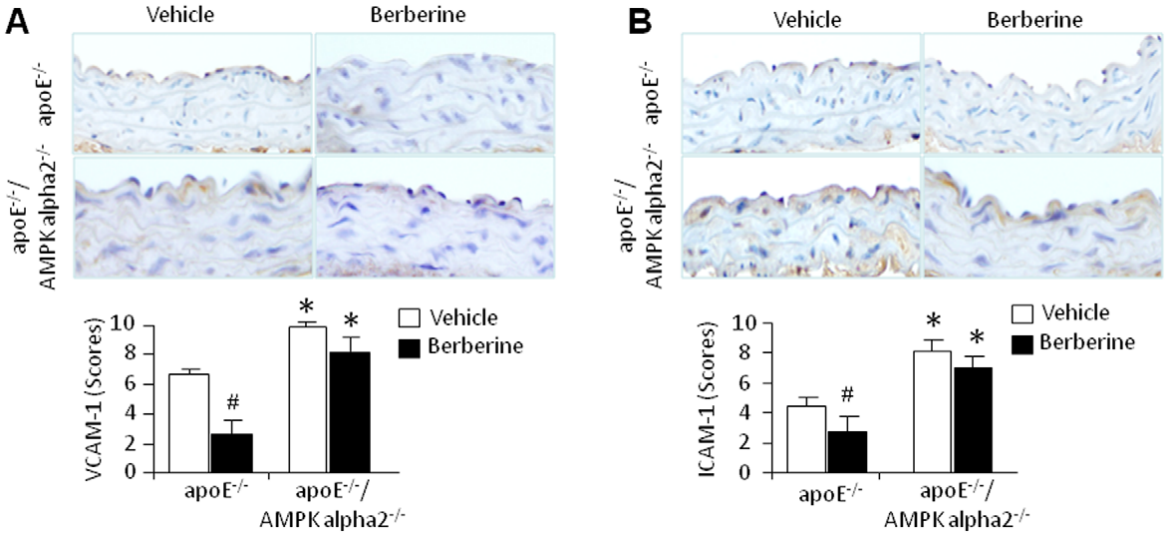
Berberine reduces VCAM-1 and ICAM-1 expression in ApoE^-/-^ and ApoE^-/-^/AMPK alpha 2^-/-^ mice. IHC was performed on sections of aortic arch atherosclerotic plaques harvested from ApoE^-/-^ and ApoE^-/-^/AMPK alpha 2^-/-^ mice fed a HFD. Sections were immunostained with antibodies specific for VCAM-1 (**A**) and ICAM-1 (**B**). *p<0.05 ApoE^-/-^ *vs*. ApoE^-/-^/AMPK alpha 2^-/-^, #p<0.05 berberine *vs*. vehicle. n = 7 or 8 for each group.

The expression of the adhesion molecules intercellular adhesion molecule-1 (ICAM-1) and vascular cell adhesion molecule-1 (VCAM-1) in endothelial cells is the earliest known event in the initiation and progression of atherosclerosis [40], [41]. To determine whether berberine might act upstream of that event, we assayed the levels of VCAM-1 and ICAM-1 in aortic lesions isolated from berberine-treated ApoE^-/-^ and ApoE^-/-^/AMPK alpha 2^-/-^ mice. Both VCAM-1 (Fig. 2A) and ICAM-1 (Fig. 2B) were highly expressed in the sections of aortic root atherosclerotic lesions harvested from ApoE^-/-^ and ApoE^-/-^/AMPK alpha 2^-/-^ mice fed the pro-atherogenic diet. However, ApoE^-/-^ animals that had been treated with berberine exhibited significant abrogation of VCAM-1 expression in aortic tissue, whereas ApoE^-/-^/AMPK alpha 2^-/-^mice did not (Fig. 2A). Berberine treatment attenuated ICAM-1 staining in ApoE^-/-^ mice, but did not affect ICAM-1 staining in the ApoE^-/-^/AMPK alpha 2^-/-^ mice (Fig. 2B).

### Berberine reduces 4-hydroxy-2-enal (4-HNE) and 3-nitrotyrsoine in aortic atherosclerotic plaques *in vivo*

**Figure 3:**
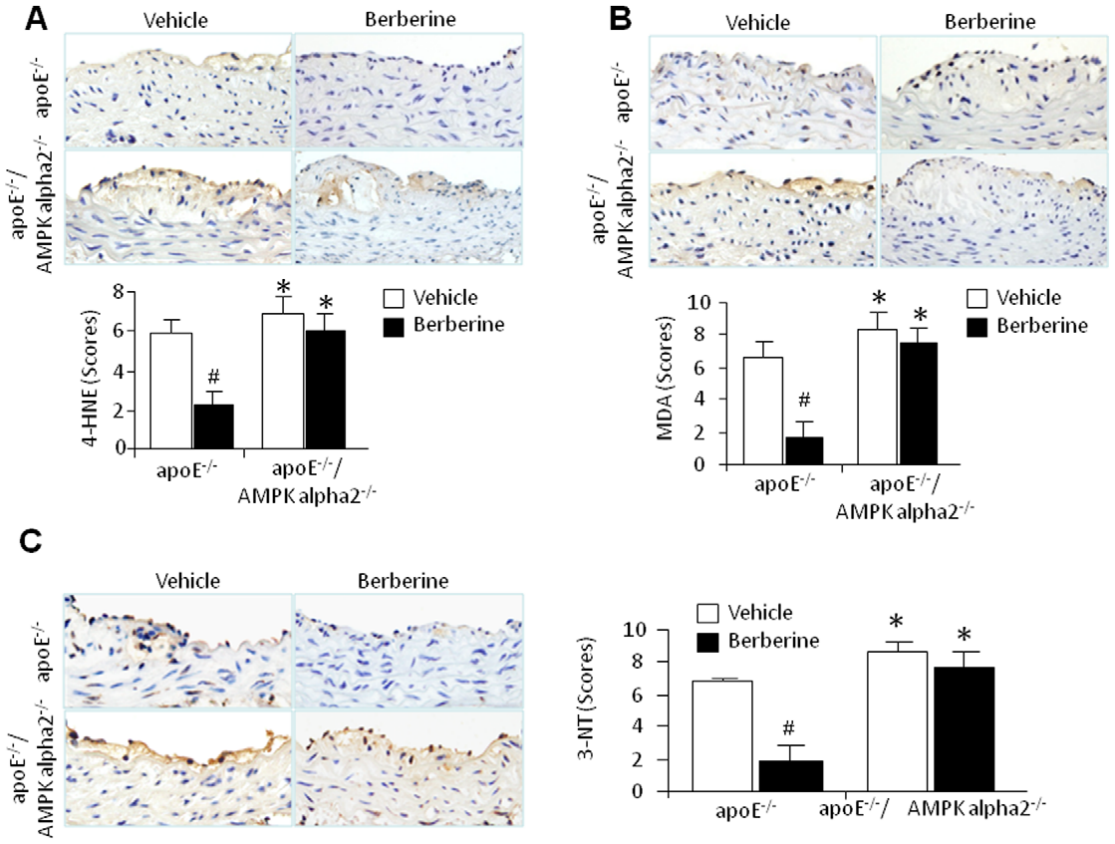
Berberine reduces oxidant stress *in vivo.* Berberine blocked expression of 4-HNE (**A**), MDA (**B**) and 3-NT (**C**) in aortic atherosclerotic plaques from ApoE^-/-^ mice but not ApoE^-/-^/AMPK alpha2^-/-^ mice. IHC was performed on sections of the aortic arch atherosclerotic plaques harvested from ApoE^-/-^ and ApoE^-/-^/AMPK alpha 2^-/-^ mice fed a HFD. Sections were immunostained with antibodies specific for 4-HNE (**A**), MDA (**B**) or 3-NT (**C**). *p<0.05 ApoE^-/-^ *vs*. ApoE^-/-^/AMPK alpha 2^-/-^, #p<0.05 berberine *vs*. vehicle. n = 7 or 8 for each group.

Oxidative damage to polyunsaturated lipids can give rise to potent cytotoxic compounds including several γ-hydroxy-α, β-unsaturated aldehydes. An important example of these aldehydes is 4-HNE, which is thought to play an important role in atherogenesis [42]. We examined whether berberine treatment affected the levels of 4-HNE in aortic atherosclerosis using IHC staining. As expected, lesions sectioned from ApoE^-/-^ animals had strong immunostaining for 4-HNE (Fig. 3A). 4-HNE staining was notably stronger in ApoE^-/-^/AMPK alpha 2^-/-^ aortas implying that the AMPK alpha 2 isoform is required to protect the endothelium from damaging effects of this aldehyde. In ApoE^-/-^ mice, but not ApoE^-/-^/AMPK alpha 2^-/-^ mice, the immunostaining for 4-HNE was significantly reduced by berberine treatment.

To further support the notion that peroxidation-based stress is involved in atherogenesis in these animals, we used IHC techniques to probe the aortic lesions for malondialdehyde (MDA), a commonly used marker of peroxidation. MDA expression was robustly evident in both ApoE^-/-^ and ApoE^-/-^/AMPK alpha 2^-/-^ lesions. Berberine markedly attenuated MDA staining in ApoE^-/-^ lesions, but not ApoE^-/-^/AMPK alpha 2^-/-^ lesions (Fig. 3B).

Nitration of protein tyrosines is considered a “footprint” of reactive nitrogen species including peroxynitrite. To further establish the effects of berberine on oxidative stress, we monitored the levels of 3-nitrotyrosine (NT)-modified proteins in aortas. Intense NT staining was found in aortas harvested from both ApoE^-/-^ and ApoE^-/-^/AMPK alpha 2^-/-^ (Fig. 3C). Berberine treatment abrogated 3-NT staining in ApoE-/- lesions but not in ApoE^-/-^/AMPK alpha 2^-/-^ lesions. These data reinforce the notion that the effects of berberine on oxidative stress rely on the AMPK alpha 2 isoform.

### Berberine-enhanced UCP2 expression is AMPK alpha 2-dependent *in vivo*

**Figure 4:**
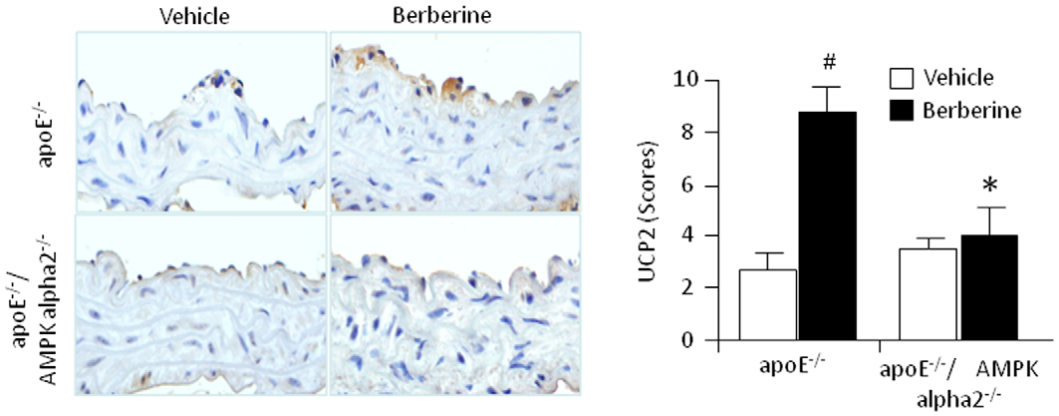
Berberine increases UCP2 expression in ApoE^-/-^ but not ApoE^-/-^/AMPK alpha 2^-/-^ mice. IHC was performed on sections of the aortic arch atherosclerotic plaques harvested from ApoE^-/-^ and ApoE^-/-^/AMPK alpha 2^-/-^ mice fed a HFD. Sections were immunostained with antibodies specific for UCP2. *p<0.05 ApoE^-/-^ *vs*. ApoE^-/-^/AMPK alpha 2^-/-^, #p<0.05 berberine *vs*. vehicle. n = 7 or 8 for each group.

Uncoupling protein 2 (UCP2) is critically involved in mitigating oxidative stress in the endothelium [43]. Earlier work from our group established that AMPK regulates the expression of UCP2 in endothelial cells [36]. Because mitochondrial uncoupling via UCP2 appears to play a critical role in rodent models of atherogenesis [36], [44], we determined whether berberine treatment up-regulates UCP2 expression. If so, that result would implicate UCP2 in the mechanism of berberine suppression of oxidative stress and vascular inflammation. UCP2 expression was almost absent in vehicle-treated ApoE^-/-^ or ApoE^-/-^/AMPK alpha 2^-/-^ mice (Fig. 4). Berberine treatment significantly increased UCP2 staining in ApoE^-/-^ lesions, but not in ApoE^-/-^/AMPK alpha 2^-/-^ lesions. Interestingly, berberine-enhanced UCP2 expression was evident in the endothelial layers and not in the sub-endothelial structures or tunica media, suggesting that the effect of berberine is relatively specific for endothelial cells and the tunica intima of the vasculature.

### Berberine increases UCP2 expression via AMPK activation

**Figure 5:**
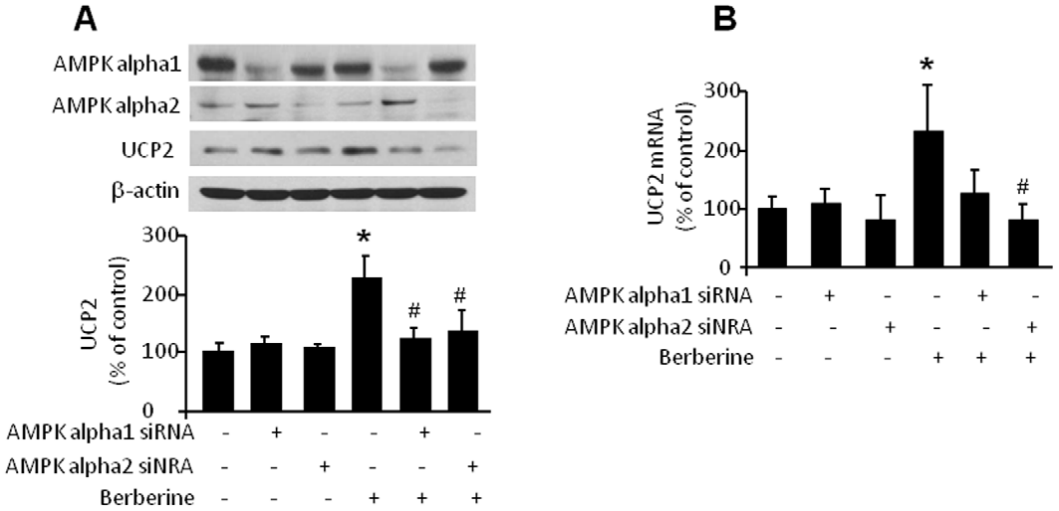
AMPK inactivation abolishes UCP2 mRNA and protein expression in HUVECs. **A.** Immunoblot analysis of UCP2 in berberine-induced HUVECs transfection with AMPK alpah1 and alpha 2 siRNA. HUVECs were transfected with control siRNA or siRNA specific to AMPK alpha 1 or AMPK alpha 2 for 48 h then stimulated by berberine (10 µmol/L) for 2 h. Cells lysates were subjected to Western blot analysis using antibody against AMPK alpha1, AMPK alpha 2, UCP2 and β-actin (n = 3; *p<0.05 vehicle *vs.* berberine treatment, # p<0.05 control siRNA *vs*. AMPK alpha 1 or alpha 2 siRNA). **B.** Quantitative PCR analysis of UCP2 mRNA in HUVECs treatment with berberine. HUVECs were infected with AMPK alpha 1, alpha 2 siRNA or control siRNA for 48hthen stimulated by berberine (10 µMol/L). Total mRNA was isolated by according to manufacturer's instructions (QIAGEN). UCP2 mRNA was quantified by real-time PCR. (n = 4; *p<0.05 vehicle *vs*. berberine treatment, # p<0.05 control siRNA *vs*. AMPK alpha 1 or alpha 2 siRNA).

Although AMPK is functionally active in several vascular cells including endothelial cells, macrophages, and vascular smooth muscle cells and berberine might exerts anti-atherosclerotic effects by suppressing the functions of macrophages and vascular smooth muscle cell, we employed endothelial cells to determine the contribution of AMPK activation to berberine induction of UCP2 expression. We performed siRNA-mediated silencing of the two AMPK alpha subunits genes, AMPK alpha 1 and AMPK alpha 2, in HUVECs. As expected, transfection of either AMPK alpha 1 siRNA or AMPK alpha 2 siRNA down-regulated AMPK alpha 1 or AMPK alpha 2 expression (Figure 5A). Importantly, transfection of either AMPK alpha 1- or AMPK alpha 2-specific siRNAs abolished berberine-induced UCP2 expression, whereas a control siRNA had no effect.

We also directly assayed UCP2 mRNA in HUVECs using real-time PCR. After treatment with berberine, the level of UCP2 mRNA was 2.4-fold higher than in untreated cells. Real-time PCR results also indicated that both AMPK alpha 1 and AMPK alpha 2 siRNAs reduced the up-regulation of UCP2 mRNA by berberine (Fig. 5B). These data imply that AMPK mediates the induction of UCP2 transcription and protein expression by berberine.

### AMPK activation increases UCP2 transcription

**Figure 6:**
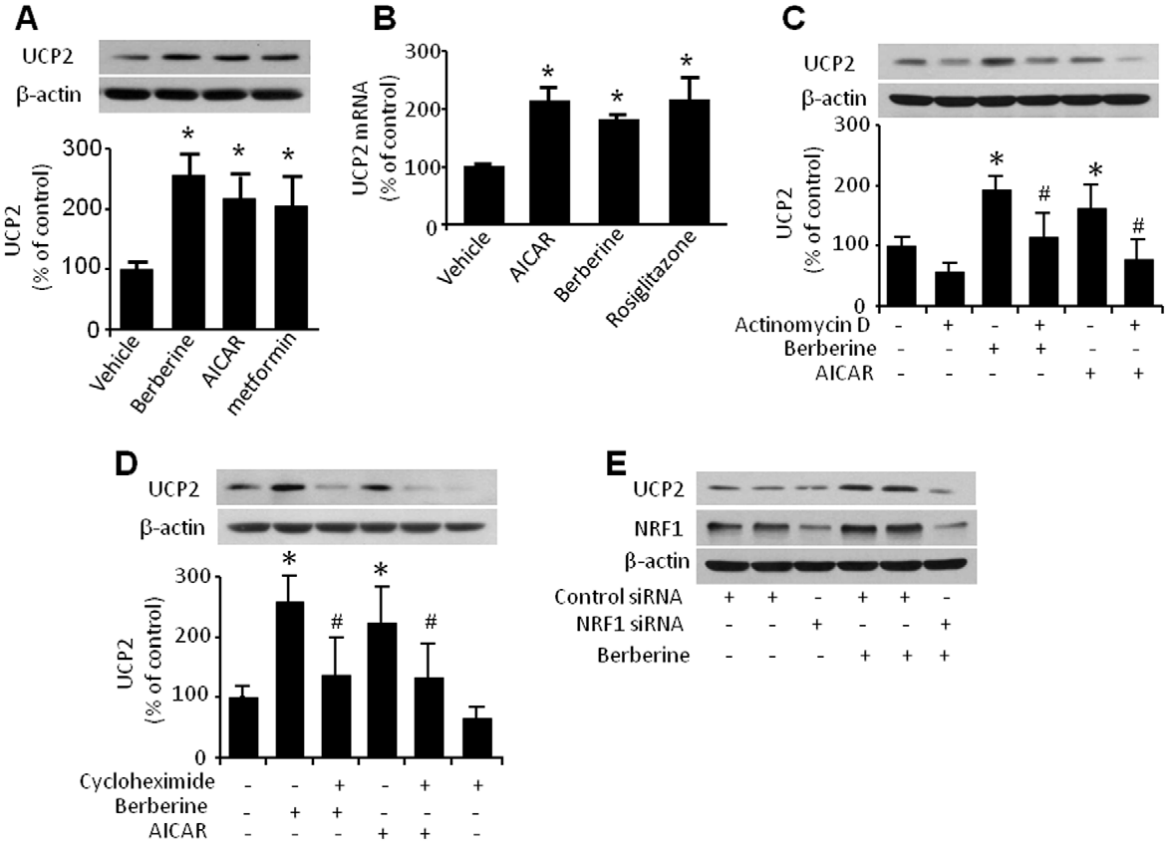
Inhibition of transcription attenuates AMPK-induced UCP2 mRNA and UCP2 protein in HUVECs. **A.** Effects of AICAR, metformin and berberine on UCP2 expression in HUVECs. n = 3; *p<0.05, vehicle *vs*. treatment. **B.** Quantitative PCR analysis of UCP2 mRNA levels in confluent HUVECs treated with AICAR (for 1 h), berberine (for 1 h) or Rosiglitazone (for 24 h). Total mRNA was isolated and UCP2 mRNA was quantified by real-time PCR compared with GAPDH. n = 4; *p<0.05, vehicle *vs*. treatment. **C.** Effect of actinomycin D on up-regulation of UCP2 by berberine. Confluent HUVECs were pretreated with actinomycin D (10 µg/ml) for 1 h, then treated with berberine or AICAR for 2 h. Cell lysates were subjected to Western blot analysis using antibody against UCP2 and β-actin. The blot is a representative of three blots from three independent experiments. **D.** Effect of cycloheximide on expression of UCP2 by berberine and AICAR. Confluent HUVECs were pretreated with cycloheximide (10 µmol/L) for 1 h, then treated with berberine or AICAR. Cell lysates were subjected to Western blot analysis using antibody against UCP2 and β-actin. (n = 3; *p<0.05 vehicle *vs*. berberine or AICAR treatment, #p<0.05 cycloheximide *vs*. vehicle). **E.** Immunoblot analysis of UCP2 expression in HUVECs transfected with NRF1 siRNA. HUVECs were transfected with control siRNA or NRF1 siRNA for 48 h, then treated with berberine for 2 h. Cell lysates were subjected to Western blot analysis using antibodies against NRF1 and UCP2. The blot is a representative of three blots from three independent experiments.

We next examined the mechanism by which AMPK activation alters UCP2 expression in cultured endothelial cells. To further define the effects of AMPK on UCP2 expression, HUVECs were incubated with several structurally unrelated AMPK activators including aminoimidazole carboxamide ribonucleotide (AICAR), metformin, and berberine. AICAR and metformin increased UCP2 expression 2-fold after 2 h treatment (Fig. 6A). Moreover, both AICAR and berberine increased UCP2 mRNA levels. Rosiglitazone, a peroxisome proliferator-activated receptors (PPARs) agonist, was employed as a positive control because it is known to increase UCP2 mRNA levels [45] (Fig. 6B). Pre-treatment of HUVECs with the transcription inhibitor actinomycin D dramatically reduced AICAR-enhanced UCP2 expression (Fig. 6C). Similarly, cycloheximide, a protein translation inhibitor, significantly inhibited both berberine- and AICAR-induced UCP2 expression (Fig. 6D). Overall, these data suggest that AMPK-dependent regulation of UCP2 expression by berberine is mediated by effects on both transcription and translation.

### NRF1 is required for AMPK-dependent upregulation of UCP2

The transcription factor NRF1 plays an important role in regulating key genes required for mitochondrial respiratory function and in promoting mitochondrial biogenesis to accompany with up-regulation of UCP2 [46], [47]. We used siRNA silencing to investigate whether NRF1 is required for AMPK-mediated UCP2 expression in HUVECs. Interestingly, we found that berberine increased NRF1 expression in the control siRNA group, suggesting that berberine regulates NRF1 (Fig. 6E). Transfection of NRF1 siRNA, but not control siRNA, ablated the up-regulation of UCP2 protein by berberine, suggesting that AMPK-dependent UCP2 expression might require NRF1.

### Berberine increases markers of mitochondrial protein synthesis and UCP2 translocation

**Figure 7:**
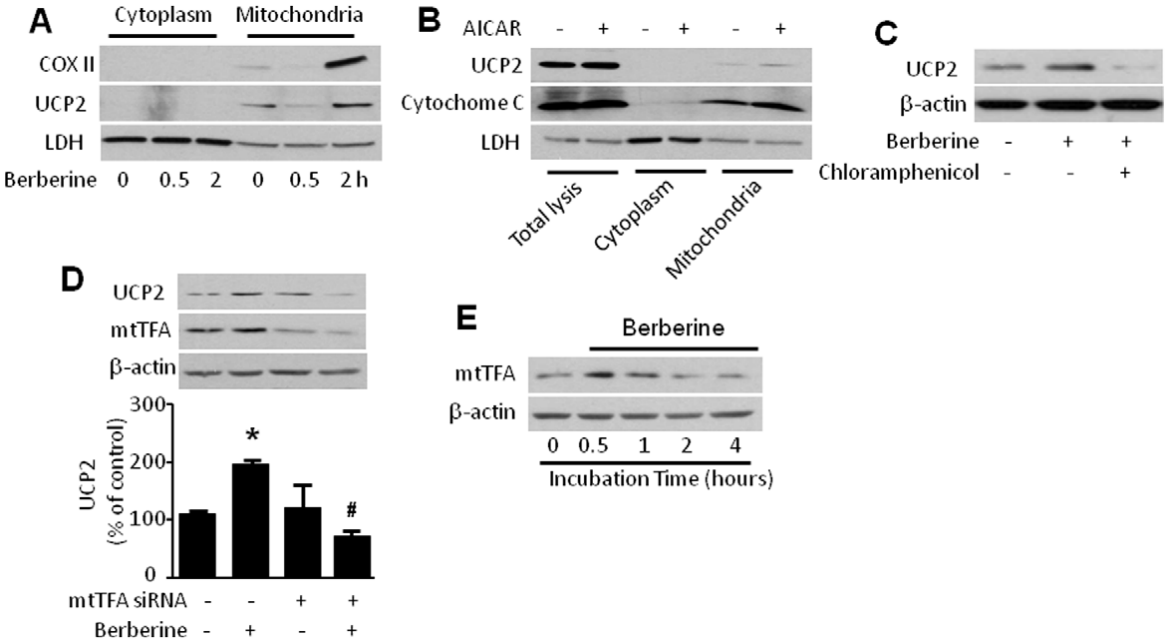
Mitochondrial biogenesis increase UCP2 expression in HUVECs treatment with berberine. **A&B.** Immunoblot analysis of mitochondrial proteins in HUVECs. Confluent HUVECs were treated with berberine (**A**) or AICAR (**B**), and the mitochondrial fraction was isolated from lysed cells using of mitochondrial isolation kit (Therm, Pockford) according to the manufacturer's instructions. A Western blot of the mitochondrial fraction was probed with antibodies specific for cytochrome C, cytochrome C oxidase II (COX II), and UCP2. The blot is representative of three blots from three independent experiments. **C.** Effect of chloramphenicol on up-regulation of UCP2 by berberine. Confluent HUVECs were pretreated with chloramphenicol (10 µmol/L), then treated with berberine for 2 h. Cell lysates were subjected to Western blot analysis using antibodies against UCP2 and β-actin. The blot is a representative of three blots from three independent experiments. **D.** Immunoblot analysis of UCP2 in berberine-induced HUVECs transfection with mtTFA siRNA. HUVECs were transfected with control siRNA or mtTFA siRNA for 48 h, then treated with berberine for 2 h. Cell lysates were subjected to Western blot analysis using antibodies against UCP2, mtTFA and β-actin. (n = 3; *p<0.05 vehicle *vs*. berberine, #p<0.05 control siRNA *vs.* mtTFA siRNA). **E.** Time course of mtTFA expression. Confluent HUVECs were treated with berberine (10 µmol/L) for the indicated times. Cell lysates were subjected to Western blot analysis using antibodies against mtTFA and β-actin. The blot is representative of three blots from three independent experiments.

The discovery that UCP2 expression was up-regulated by berberine treatment led us to examine whether other critical mitochondrial proteins might also be affected by berberine. We therefore investigated whether berberine increased the synthesis of other mitochondrial proteins in HUVECs by comparing protein levels in mitochondrial and cytoplasmic fractions. Berberine-induced UCP2 expression was observed in the mitochondrial fraction but not in the cytoplasm, as expected (Fig. 7A). Berberine also increased the expression of cytochrome C oxidase, which is encoded in mitochondrial DNA and induced by mtTFA, a mitochondria-specific transcription factor. The AMPK activator AICAR also increased cytochrome C oxidase and UCP2 expression in the mitochondrial fraction (Fig. 7B). This set of data suggests that berberine stimulates mitochondrial biogenesis via AMPK activation.

We also examined whether mitochondrial biogenesis might promote the apparent upregulation of UCP2 protein levels in the mitochondria by increasing UCP2 translocation from the nucleus, where it is encoded, to the mitochondrion where it acts. Pre-treatment of HUVECs with chloramphenicol, an inhibitor of mitochondrial DNA transcription, markedly reduced berberine-induced UCP2 expression (Fig. 7C). This finding lends strength to the notion that UCP2 translocation was inhibited in the HUVEC assay.

mtTFA is a known mitochondrial transcription factor that controls mitochondrial DNA transcription [48]. Transfection of HUVECs with mtTFA-targeted siRNA, but not control siRNA, dramatically reduced the berberine-induced increase in UCP2 expression (Fig. 7D). Also, berberine induction of mtTFA expression was observed as early as 30 min after berberine treatment, suggesting that mitochondrial biogenesis is promoted by berberine (Fig. 7E).

## Discussion

The salient and novel finding of this study is that berberine appears to manifest its anti-atherogenic properties via AMPK-dependent ROS suppression, likely via increased expression of UCP-2. Further, we found that AMPK-enhanced UCP2 expression was mediated by NRF1. This finding is consistent with our recent report that the α2 subunit of AMPK is critical for mitigating ER stress and protecting from atherosclerosis *in vivo* [49]. Our results are in line with previous reports that AMPK activation by S17834, a polyphenol, suppresses atherosclerosis caused by diabetes in LDL receptor (LDLr) knockout mice [50] and that ApoE^-/-^/AMPK alpha 2^-/-^ mice display accelerated development of high-fat diet-induced aortic lesions.

UCP2 is a mitochondrial transporter present in the inner membrane of mitochondria and belongs to the family of anion mitochondrial carriers. The absence of UCP2 in mice has been reported to increase oxidant stress [51], [52] and to amplify the development of atherosclerotic plaques [53]. Increasing evidence, summarized below, supports a role for UCP2 as a modulator of mitochondria-derived reactive oxygen species (ROS) [54], [55]. UCP2 is reported to reduce ROS not only in the mitochondria, but within the whole cell and even in the extracellular space [43], [56], [57]. A recent report indicates that UCP2-mediated uncoupling in endothelial cells can decrease extracellular ROS when co-incubated with low-density-lipoproteins (LDL) [43], [58]. In leptin-deficient *ob*/*ob* mice, diminished UCP2 levels and increased mitochondrial ROS production relative to normal mice are found in conjunction in macrophages [59]. UCP2^-/-^ mice [52] and UCP3^-/-^ mice [60] exhibited higher levels of ROS in macrophages and muscle, respectively. Furthermore, mice with deleted LDL receptor exhibited extensive HFD-induced atherosclerotic plaques when they received bone marrow transplanted from UCP2^-/-^ mice, and that phenotype was not observed when they received bone marrow transplants from UCP2^+/+^ mice [53]. Thus, these findings support a key role for UCP2 in mice in mitigating oxidant stress and inhibiting pathways that lead to the development of atherosclerotic plaque [44].

We consistently found increased UCP2 expression associated with decreased levels of oxidative stress markers in berberine-treated ApoE^-/-^ mice. Conversely, decreased UCP2 expression was observed to parallel increased inflammation and oxidative stress. Indeed, ApoE^-/-^/AMPK alpha 2^-/-^ mice exhibit enhanced oxidative stress with defective UCP2 expression. Accelerated aortic lesions in ApoE^-/-^/AMPK alpha 2^-/-^ mice are suppressed by the antioxidant tempol (a cell-permeable nitroxide free radical that acts as a radicalscavenger and nitric oxide spin trap) [49], indicating a causative role of ROS in aortic lesions in ApoE^-/-^/AMPK alpha 2^-/-^ mice. Thus, it is reasonable to speculate that increased expression and activation of UCP2 by berberine play a major factor in mitigating aortic lesions *in vivo*. The suggestive experiments with cultured cells described here do not address directly whether berberine-enhanced UCP2 expression can reduce oxidative stress and atherosclerosis *in vivo*. Thus, further investigation is needed to demonstrate this hypothesized *in vivo* role of UCP2.

Our published data [31] have demonstrated that production of ROS is responsible for berberine-induced AMPK activation, which is seemingly in conflict with the inhibitory effects of berberine on ROS reported here. This apparent conflict might be resolved by the concept of “ischemic preconditioning”, in which suboptimal concentrations of ROS activate the expression of antioxidant genes or suppress the expression or activity of oxidant-producing enzymes. For example, our published reports showed that AMPK can suppress ROS production by suppressing NAD(P)H oxidase [61] and increasing expression of antioxidant genes such as MnSOD or UCP2 [36]. Recent evidence indicating that AMPK activation reduces hyperglycemia-induced mitochondrial ROS production was provided by Kukidome et al., who showed that induction of MnSOD and promotion of mitochondrial biogenesis occurs via activation of the AMPK-PGC1α pathway in HUVECs [62]. Interestingly, Brand's group promoted the idea that O_2_^-^ itself activates UCP-2 by unspecified mechanisms from the matrix side of the mitochondrion [63]. Although the molecular mechanisms might be complex, it is reasonable to hypothesize that a ROS-AMPK-UCP2 axis might be keys to maintaining vascular redox homeostasis and functions.

In summary, we have demonstrated that berberine limits aortic lesions *in vivo* via AMPK activation. In addition, the reduction of aortic lesions by berberine appears to be mediated by enhanced expression of UCP2, which suppresses oxidative stress and vascular inflammation. The data presented here support the concept that AMPK may be an important therapeutic target for treating atherosclorosis and other cardiovascular diseases.
